# Effects of plastic fragments on plant performance are mediated by soil properties and drought

**DOI:** 10.1038/s41598-022-22270-5

**Published:** 2022-10-22

**Authors:** Anne Krehl, Undine Schöllkopf, Maria Májeková, Katja Tielbörger, Sara Tomiolo

**Affiliations:** Plant Ecology Group, Institute for Evolution and Ecology, Tübingen University, Auf der Morgenstelle 5, 72076 Tübingen, Germany

**Keywords:** Climate-change ecology, Population dynamics, Ecology, Evolutionary ecology

## Abstract

In recent years, the effects of plastic contamination on soil and plants have received growing attention. Plastic can affect soil water content and thus may interact with the effects of drought on soil and plants. However, the effects of plastic on soil are highly context-dependent, and interactions with drought have been hardly tested. We conducted two greenhouse experiments to test the combined effects of plastic fragments (of varying size and concentration), water availability and soil texture, on soil water content and performance of the plant *Arabidopsis thaliana*. Plastic fragments had stronger negative effects on soil water content in low water availability, and the shape of this response (linear vs. unimodal) was mediated by soil texture. Conversely, increasing concentration of plastic had positive effects on plant growth. We suggest that plastic fragments introduce fracture points within soil aggregates. This increases number and size of soil pores favoring water loss but also facilitating root growth. Our results suggest complex interactive effects of plastic and drought, that may lead to a decoupling of plant and soil response. These processes should be taken into account in ecological studies and agricultural practices.

## Introduction

A major focus of ecological research is the study of plant and soil responses to global change factors^1,2^. While some of these factors, such as climate change, have been the focus of much research on plant communities, other novel stressors of anthropogenic origin have emerged only recently. Among those is plastic contamination in terrestrial environments^3,4^. Although the number of studies addressing the effect of plastic contamination on ecological communities is increasing, we have virtually no knowledge about the potential interactive effects of climate change factors, such as drought, and plastic contamination on soil and plants. This knowledge is urgently needed for understanding global change effects on natural and agricultural ecosystems.

Since the 1950s, a steadily increasing amount of plastic waste has been introduced into the environment. The estimated global annual production of plastic has exceeded 300 million metric tons^5,6^, and it is predicted to increase. Due to its high durability, plastic waste persists in the environment for a long time^4^, during which it breaks into in smaller particles largely variable in size (centimeters to micrometers), shape, and behavior^7–9^.

Although much of the research on the effect of plastic contamination on the environment has focused on aquatic systems^3^, terrestrial ecosystems have been signaled as ‘major sinks’ of plastic debris^10,11^. A major input of plastic into terrestrial environments comes from agricultural practices, where the use of mulching foils is extensive^12–14^. Mulching foils are large plastic sheets (chiefly composed of polyethylene) that are applied on the soil to reduce evaporation, stabilize temperature, hinder the growth of weeds, and optimize crop germination^12,14,15^. Though mulching foils are usually removed upon germination, they often break apart and leave behind many smaller fragments that are spread across the landscape by wind and runoff^11,16^ and are incorporated into deeper soil horizons by soil organisms^17,18^. In farmlands, the annual consumption of mulching foils varies between 5 and 35 kg ha^−1^, amounting to plastic residuals in the range of 72–260 kg ha^−1^^19^. Thus, areas devoted to agricultural practices are potential hotspots of plastic contamination from which plastic debris can be dispersed into adjacent natural communities.

Experimental studies indicate that plastic fragments affect several soil properties, such as soil porosity and soil water content^20–22^. Although such effects vary in strength and direction depending on the properties of plastic fragments (e.g. shape, size, concentration and chemical composition)^23^, studies have indicated that higher concentration and larger sizes of plastic fragments are associated with lower soil water content due to either increased evaporation or percolation^21,24,25^. When incorporated into soil aggregates, plastic fragments favor the formation of fracture lines and reduce soil aggregate stability^9,20,24–26^, thus promoting the formation of soil pores along which water can move vertically. Therefore, plastic can either augment water loss when facilitating the formation of larger soil pores through which water flows faster, or reduce water loss, when promoting the formation of small pores through which water moves slowly due to capillarity^27^.

Finally, soil texture might mediate the effects of plastic on soil water content, due to inherent differences in water retention between sand- versus clay-rich soils. Soil texture determines the spatial arrangement of soil aggregates and pore networks, thus affecting soil water content and the movement of water and nutrients in soils^27,28^. Under optimal watering conditions, in clay-rich soils, the presence of aggregates favors higher water holding capacity compared to sand-rich soils, where water percolates faster through loose soil grains. However, under drought the situation is reversed, clay-rich soils form larger aggregates, leading to wider soil pores and faster water loss compared to sand-rich soils^29,30^. Given that plastic fragments affect the stability of soil aggregates, their effects are likely to be larger on clay-rich soils than on sand-rich soils. Thus, the combined effects of plastic fragments and drought on soil water content may be mediated by soil texture (e.g. clay vs. sand content), giving rise to high context-dependence in such interactions.

Soil structure and soil water content are known to affect plant growth and performance^31–33^, and plastic fragments may influence such responses to an extent that is so far not fully understood^34^. On the one hand, plastic-mediated shifts in soil water content can either mitigate or amplify the effects of drought on plants. On the other hand, plastic fragments may facilitate plant growth by increasing the size and number of soil pores through which roots can grow, thus attenuating the negative effects of soil water content decline. In the context of increasing climate-change-induced droughts and raising levels of plastic waste production, the combined effects of plastic and drought on soil and plants may have long-term consequences on plant species and communities^35^.

**Figure 1:**
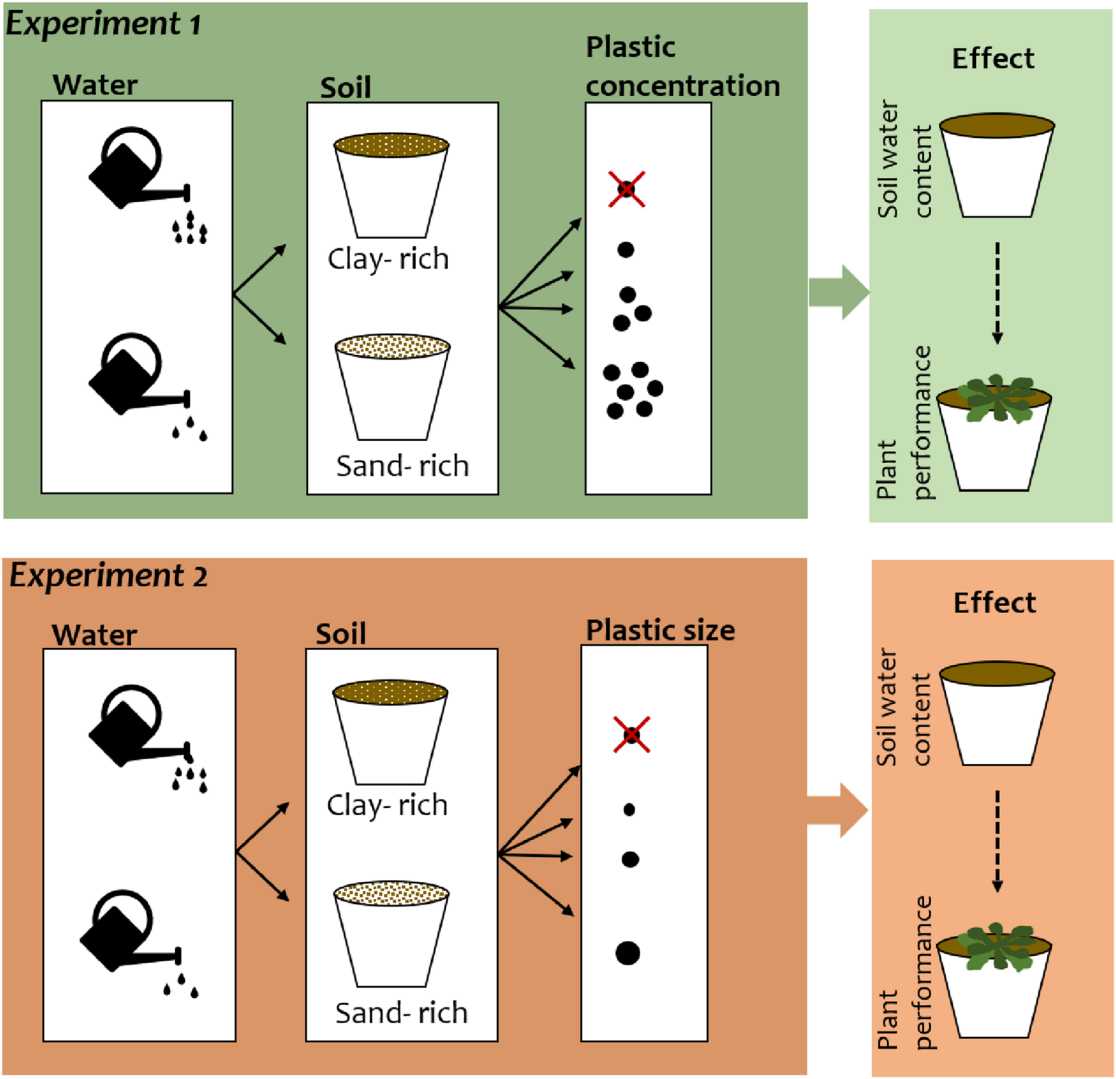
Scheme of the two experiments, where we tested how soil water content and plant performance respond to different levels of water availability (low vs. high), soil texture (clay-rich vs. sand-rich) and plastic concentration (*Experiment 1*) or plastic size (*Experiment 2*).

We present the results of two greenhouse experiments (Fig. 1), where we tested the response of soil water content and plant performance to the full-factorial combination of two water treatments (optimal, 75% soil saturation, vs. reduced, 30% soil saturation) and two soil textures (clay-rich vs. sand-rich), which were exposed to either increasing concentration of plastic fragments (0%, 0.1%, 0.5%, 1% w/w) in Experiment 1, or increasing size of plastic fragments(0, 4 mm, 6 mm, 8 mm) in Experiment 2. Plant growth of the model plant *Arabidopsis thaliana* was measured over the span of nine weeks, and soil water content was measured using pot weight as a proxy, in pots containing only soil as well as in planted pots. We hypothesized that (1) the presence of plastic fragments leads to a decreased soil water content, and this effect is more pronounced (a) when plastic fragments are present in higher concentrations or larger sizes, (b) in clay-rich soils compared to sand-rich soils, (c) in low compared to high water treatments; (2) plastic fragments have an indirect effect on plant performance, which is mediated by soil water content. Namely, plant performance is lower in treatments combinations where we expect soil water content to be reduced.

## Results

We obtained data on plant radial and vertical growth, above- and below-ground biomass, fruit production of *A. thaliana* plants (n = 160 pots for each experiment), as well as pot weight used as proxy for soil water content (n = 160 pots for each experiment). Measurements were recorded weekly.

### Effect of plastic fragments concentration and size on soil water content

#### Experiment 1: effects of plastic fragments concentration

**Figure 2:**
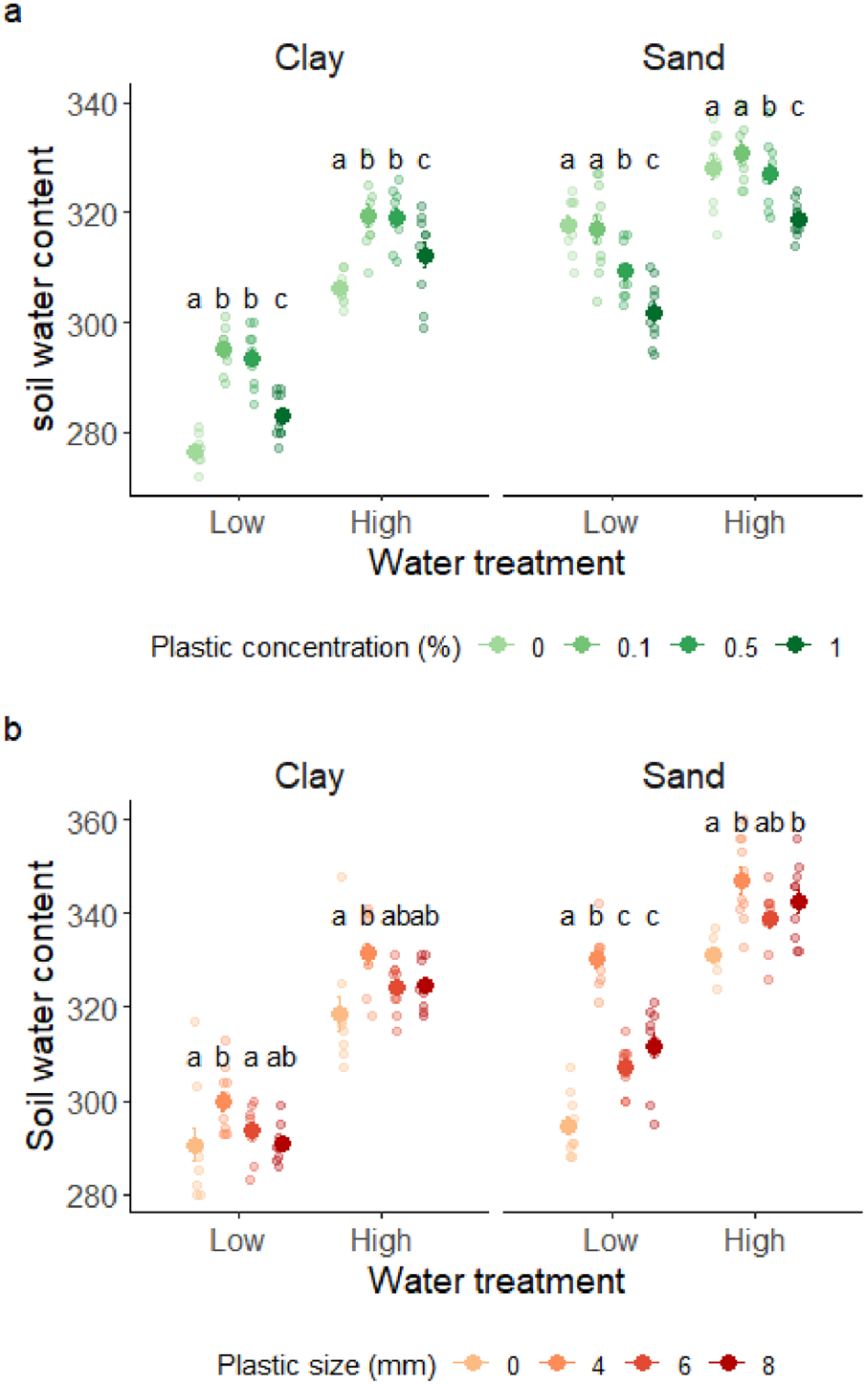
Plots showing mean ± 1SE soil water content (for which pot weight was used as a proxy) of unplanted pots, as a function of water treatment, soil texture and plastic fragments’ concentration (**a**) or size (**b**). Results of significant differences (*p* < 0.05) for pairwise comparisons of the effects of plastic fragments within each combination of soil texture and water treatment are reported with different letters. Semi-transparent dots represent the raw data. Lower pot weight represents a proxy for lower soil water content.

We found significant interactions between soil texture and watering treatment, and between soil texture and plastic concentration (Table 1, Fig. 2a). The mean difference in soil water content between water treatments was twice as large in clay-rich soil compared to sand-rich soil. In clay-rich soils we found a hump-shaped relationship between plastic concentration and soil water content in both water treatments. Soil water content was lowest in control treatments (no plastic) and highest in the intermediate plastic concentrations (0.1% and 0.5% w/w, respectively 1 g/kg and 5 g/kg), with the highest plastic concentration (1% w/w, corresponding to 10 g/kg) in between. On the contrary, in sand-rich soils, the effect of plastic concentrations on soil water content decreased with increasing plastic concentrations in both low- and high-water treatments. Analyses of soil water content for planted pots revealed results largely consistent with soil water content of unplanted pots (Supplementary Information, Table S1, Fig. S2a). The only exception was clay-rich soil pots receiving high water treatment, where soil water content decreased with increasing plastic concentration.

**Table 1:** Results of linear models for *Experiment 1*, testing the effects of water treatment (‘watering’), soil texture (‘soil’) and concentration of plastic fragments (‘plast_conc’) on soil water content of unplanted pots and radial growth of *A. thaliana* plants.

Model terms	Soil water content			Radial growth		
	df	F. test	*p*. value	df	F. test	*p*. value
Soil	1	295.10	**< 0.001**	1	18.26	**< 0.001**
Watering	1	18.80	**< 0.001**	1	243.93	**< 0.001**
Plast_conc	3	19.35	**< 0.001**	3	15.72	**< 0.001**
Soil:watering	1	32.72	**< 0.001**	1	7.36	**0.008**
Soil:plast_conc	3	22.86	**< 0.001**	3	3.28	**0.023**
Watering:plast_conc	3	1.96	0.122	3	2.10	0.103
Soil:watering:plast_conc	3	2.19	0.092	3	2.04	0.111

Significant effects (*p* < 0.05) are reported in bold.

#### Experiment 2: effects of plastic fragments size

We found a significant three-way interaction among soil texture, plastic size and watering treatment (Table 2, Fig. 2b). In both water treatments and across soil textures, the highest level of soil water content was found in pots with plastic fragments size 4 mm. In clay-rich soil, soil water content of pots treated with larger plastic fragments (6 mm and 8 mm) was not significantly different from control pots, regardless of water treatment. In sand-rich soil, soil water content in control pots was significantly lower (between 3 and 7%) compared to plastic-treated pots. Such effect was more pronounced in low- water treatments (up to 11% lower soil water content in control than in plastic pots) compared to high-water treatments (up to 5% lower soil water content in control pots). Results of pot weight for planted pots were very similar to those on unplanted pots, with the exception of clay-rich soil in low water treatments, where soil water content increased with plastic size (Supplementary Information, Table S2, Fig. S2b).

**Table 2:** Results of linear models for *Experiment 2*, testing the effects of water treatment (‘watering’), soil texture (‘soil’) and size of plastic fragments (‘plast_size’) on soil water content of unplanted pots and radial growth of *A. thaliana* plants.

Model terms	Soil water content			Radial growth		
	df	F. test	*p*. value	df	F. test	*p*. value
Soil	1	15.17	**< 0.001**	1	6.00	**0.016**
Watering	1	76.64	**< 0.001**	1	481.75	**< 0.001**
Plast_size	3	5.25	**0.002**	3	0.62	0.603
Soil:watering	1	3.42	0.066	1	0.23	0.632
Soil:plast_size	3	0.51	0.674	3	0.37	0.772
Watering:plast_size	3	0.54	0.656	3	0.16	0.924
Soil:watering:plast_size	3	4.50	**0.005**	3	1.07	0.366

Significant effects (*p* < 0.05) are reported in bold.

### Effect of plastic fragments concentration and size on plants

In both experiments, radial growth, above- and below-ground biomass, and plant height were strongly positively correlated (as shown in the correlation matrix in Supplementary Information, Fig. S3). Additionally, root-to-total ratio and number of fruits showed limited responses to the experimental factors and were not affected by plastic treatments. Thus, we present the results for radial growth only, while the other results are shown in the Supplementary Information, Appendix 1.

#### Experiment 1: effect of plastic fragments concentration

**Figure 3:**
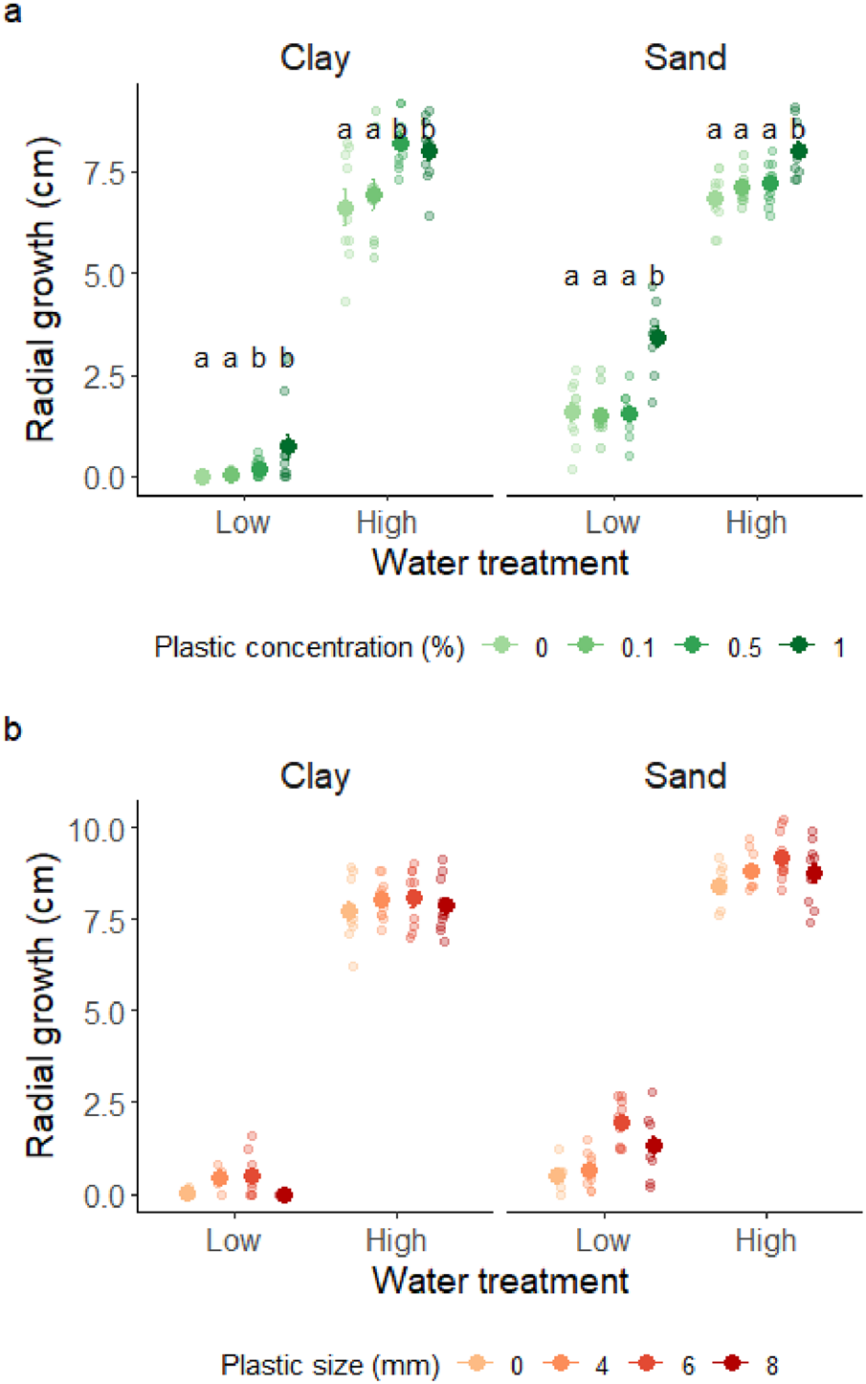
Plots showing mean ± 1SE radial growth of *A. thaliana* as a function of water treatment, soil texture and plastic fragments’ concentration (**a**) or size (**b**). Results of significant differences (*p* < 0.05) for pairwise comparisons of the effects of plastic fragments within each combination of soil texture and water treatment are reported with different letters. Semi-transparent dots represent the raw data.

We found significant two-way interactions between soil texture and water treatment, and between soil texture and plastic fragments concentration (Table 1, Fig. 3a). In both soil textures, plant growth was significantly higher in high water treatments. The positive effects of high watering on plant growth were in average 35% higher in clay-rich compared to sand-rich soil.

In clay-rich soil, growth was consistently higher in plastic concentrations of 0.5% (5 g/kg) and 1% (10 g/kg) compared to the lowest concentration 0.1% and control treatments with no plastic. In sand-rich soil, plants attained the highest radial growth when exposed to the highest concentration of plastic fragments (i.e. 1%), compared to lower plastic concentrations and control treatments.

#### Experiment 2: effect of plastic fragments size

When looking at the full dataset, we found that the effect of the watering treatments was highly significant (Table 2, Fig. 3b), and its effect size was between 10 and 20 times stronger than the effects of soil texture and plastic size. To check whether the strong effect size of watering treatments masked smaller, yet potentially important, effects of soil texture and plastic size, we split the data across watering treatments, and tested the combined effects of plastic size and soil texture on plant growth. In low watering treatments, we found a significant interaction between soil texture and plastic size (F_3_ = 4.07, *p* = 0.012, Supplementary Information, Fig. S4a), whereby in sand-rich soil plant growth was higher when plants were exposed to plastic fragments of size 6 mm and 8 mm (the two larger sizes) compared to control treatments (no plastic added) and plastic fragments of size 4 mm (the smallest fragments), whereas in clay-rich soil no significant effect of plastic fragments was detected. In high watering treatments, plant growth was in average 10% higher in sand-rich soil compared to clay-rich soil (F_1_ = 32.02, *p* < 0.001, Supplementary Information, Fig. S4b), but no significant effect of plastic fragments was detected.

## Discussion

Our overall results indicate that soil texture modulated the effects of plastic fragments both on soil and plants, and that at least some of the responses were amplified under low water availability. Contrary to our expectations, plastic fragments did not always have a negative effect on soil water content. Soil texture mediated the shape, rather than the strength, of plastic effects on soil water content, and the response of plants did not follow the same pattern as soil water content. In the following, we discuss our findings with respect to our initial hypotheses.

Soil texture did not modulate the strength of soil response to plastic treatments, but it mediated different response patterns to plastic concentration, where in clay-rich soils we observed a hump-shaped response and in sand-rich soils a linear response. These results suggest the importance of accounting not only for different levels of plastic contamination, but also for different soil texture.

Increasing plastic concentration and size were associated with higher soil water loss. However, with the exception of sand-rich soils in *Experiment 1*, control treatments had consistently the lowest levels of soil water content. Increased soil water content in low concentrations of plastic fragments, was also found for different types of plastic materials in a previous study^9^, but was not detected for polyethylene fragments^24^. Previous studies have suggested that high concentration and size of plastic fragments may favor soil water loss by means of hindering the process of soil aggregation^20,25^ and contributing to an increased number and size of soil pores through which water percolates or evaporates. In *Experiment 1*, which manipulated plastic fragments’ concentration, the positive effect of low plastic concentrations on soil water content observed in clay-rich soil, may be explained by the fact that, when in small concentrations, plastic fragments increased soil porosity and ameliorated soil water retention. Whereas increasing concentrations produced the opposite outcome, that is, larger and more numerous pores along which water percolation or evaporation was enhanced. In sand-rich soils, increasing concentrations of plastic may have led to larger channels among soil particles, thus accelerating water loss. For *Experiment 2*, in which we manipulated plastic size while keeping concentration constant at 0.1% (the lowest concentration represented in Experiment 1), the overall positive effect of plastic on soil water content was consistent across soil textures. Our data might not provide a satisfactory explanation for the different response to plastic across soil textures in the two experiments, as in this study we focused on the physical effects of plastic on soil properties. However, other studies have showed that plastic affects among others soil enzymatic activity^25^, soil pH^21^ and soil microbes^40,45^. The simultaneous effects of plastic fragments on multiple soil properties have been put forward as a possible reason for the non-linear responses of soil to increasing size and concentration of plastic observed in previous research^21^. To date, however, it is not yet possible to fully disentangle such interactions due to the high complexity of soil systems.

In general, the effect of plastic treatments on soil water content was stronger in low water treatments, suggesting that under drought the effects of plastic (either positive or negative) on soil water content may become more drastic, and that studies investigating the effects of plastic in soil under optimal watering regime, may overlook some important effects. Drought, similarly to plastic contamination, affects soil aggregation and wettability^36^, but while the effects of drought on soil water content were always negative, those of plastic varied across plastic properties (i.e. concentration and size) and soil texture. Thus, the outcome of interactions between drought and plastic contamination is likely to be highly context dependent. Additionally, research has highlighted that drought and plastic alter soil microbial communities in substantially different ways^37–40^, as the first was associated to an increase in Gram positive versus Gram negative and the second to the opposite pattern. Although our experiment focused solely on soil water content and on the physical aspect of plastic fragments, future research should endeavor to investigate the effects of plastic contamination on chemical and physical soil properties and soil biota, under varying levels of water availability.

While the effect of soil texture mediated in some cases the pattern of soil and plant response to plastic contamination, watering treatment modulated the strength of this response. This introduces a series of methodological considerations. First, accounting for soil texture is essential to understanding if and how patterns of plant and soil responses to plastic contamination may vary across soils, and may help reconcile several contrasting results in the existing literature (e.g.^9,25^), where experiments were conducted using one type of substrate. Second, water availability appeared to have a stronger effect than plastic contamination in our experiments, and this was particularly the case in *Experiment 2*, where we applied the lowest of the concentrations of plastic fragments used in this study (1 g/kg). While this concentration corresponds to high levels of contamination for natural areas, and possibly also for agroecosystems^48–50^, we cannot exclude that such levels of contamination may be reached or surpassed in a not so distant future, given the increasing accumulation of plastic in the environment^6^. Thus, it would be advisable that future studies consider how optimal levels of watering may mask the subtle, yet present, effects of plastic contamination on soil water content and plant growth.

Plants grew more when exposed to the higher concentrations of plastic fragments and, to a smaller extent, to larger plastic sizes. Namely, plants grew more in combinations of soil texture and plastic fragments where soil water content was found to be the lowest. This is opposite to previous results showing negative impacts of plastic on plant growth^41,42^. De Souza Machado et al.^9^ found increased root biomass in plastic-treated soil, which could be indication of plant response to increased water stress. However, in our study we did not observe shifts in root allocation in plastic-treated versus control pots. Instead, consistently with previous studies, our results indicate that plastic may have indirectly facilitated plant growth by increasing soil pores through which plant roots could develop and grow^43,44^. Additionally, the presence of polyethylene film fragments in soil was associated with increased abundance of Proteobacteria^45^, a group of plant-growth promoting bacteria^46,47^. These results are important because they suggest a possible decoupling in the response of soil and plants to plastic fragments, thus making predictions of potential responses of plant-soil systems even more challenging. As this study was conducted over nine weeks, the response of plant to plastic contamination and drought may differ when considered over a longer temporal scale and when accounting for interactions between plants and soil microbes. For example, positive effects of plastic on root growth may be negligible when considering the effects of prolonged drought and sustained inputs of plastic debris on soil properties. Lastly, while drought and plastic may affect plants indirectly via shifts on soil water content and soil biota, plants in turn affect soil abiotic properties (e.g. soil aggregation) and modify soil biota via plant-soil feedback, and may possibly mitigate some of the effects of plastic on soil aggregation^26^.

Our results present a proof of concept of the potential combined effects of water availability and plastic contamination on soil properties and plant performance. Specifically, soil texture and water availability mediated respectively the shape and the strength of plastic fragments’ effects on soil water content. Additionally, the discrepancy among plant and soil responses to plastic treatments suggests a potential decoupling in the effects of this stressor. Taking into consideration the results of the current study, as well as those of previous research, we argue that the complexity and high context-dependence associated to soil and plants responses to plastic contamination and drought, makes predictions and generalizations difficult. As such, future studies should focus on integrating these two stressors and their potential effects on soil abiotic and biotic properties, as well as plant-soil biota interactions, to better understand how natural and semi-natural communities may cope with these stressors.

## Material and methods

To test if water availability and soil texture mediate the effects of plastic contamination on soil water content and plant performance, we set up two greenhouse experiments where we added plastic fragments in increasing concentration (*Experiment 1*) and increasing size (*Experiment 2*) to two soils differing in texture, and exposed them to two different watering treatments (Fig. 1).

*Substrate preparation* To mimic different soil textures, we prepared two soil substrates by mixing sand and clay in different proportions. The sand-rich soil was obtained by mixing 70% sand and 30% clay in volume (estimated soil density: 1.59 g/cm^3^), and the clay-rich soil was obtained by reversing the ratios and mixing 70% clay and 30% sand in volume (estimated soil density: 1.44 g/cm^3^). The sand, which was purchased from the German company Flammer Bauunternehmung GmbH, originated from the Rhine river banks. The clay was purchased from the company Sand- und Kieselwerk Matthäus Bischoff GmbH & Co. KG. Before preparing the two soil mixtures, we autoclaved the sand for five hours at of 110 °C, and we reduced the size of the clay aggregates first processing it through a soil shredder and then sieving it through a 5 mm mesh sieve. Subsequently, sand and clay were mixed in the prescribed proportions using a cement mixer. After mixing, the soil was checked again for large aggregates to guarantee an even texture.

*Plastic fragments* To produce plastic fragments, we used a black low-density polyethylene (LDPE) mulching film (IsolenTM Premium Schwarz produced by Barbier Group, www.barbiergroup.com), with density between 0.915 and 0.935 g/cm^3^ and thickness of 30 µm (as per product specification). Folded foils were perforated with a round hollow punch to produce fragments that were homogeneous in shape (circular) and varied in diameter: 4 mm, 6 mm and 8 mm (Supplementary Information Fig. S1). Fragments were subsequently inspected to separate multiple layers of film, when found stuck together. Plastic fragments were stored in previously sterilized glass jars for approximately three weeks. For *Experiment 1*, in which we manipulated the concentration of plastic fragments, we used plastic fragments 6 mm in size and mixed them with soil in four different concentrations of 0 g/kg (control treatment), 1 g/kg (0.1% w/w), 5 g/kg (0.5% w/w) and 10 g/kg (1% w/w). These concentrations are consistent with environmentally relevant estimates under different levels of contamination^48–50^. For *Experiment 2*, in which we manipulated the size of plastic fragments, we used fragments in concentration of 1 g/kg and mixed them with soil using the three different sizes produced (4 mm, 6 mm, 8 mm), and a control treatment where no plastic fragments were added. Plastic fragments were evenly incorporated into the two different soil substrates, and subsequently the different combinations of soil and plastic fragments were distributed into 0.3 l pots (diameter 9 cm, height 7 cm). Control soils were mixed as well to account for the effect of soil mixing.

*Watering treatments* We applied two watering treatments to simulate high water availability and drought. Pots assigned to the high water treatment received an amount in water corresponding to 75% of the mean pot saturation weight, whereas pots assigned to the low water treatment received an amount of water corresponding to 30% of mean pot saturation weight. Pots were watered three times a week to maintain a stable water level in soil and avoid pulses. To ensure that the amount of water added corresponded to the percentage of soil saturation weight assigned to each treatment, pots were weight weekly and watering amount adjusted when necessary. Because water holding capacity changes depending on the clay: sand ratio, the actual watering amount may change with soil texture and overtime, but was kept constant across plastic treatments within each combination of soil texture and watering by means of weighting the pots weekly.

To disentangle the effects of plastic fragments on soil water content from those on plant performance, we prepared pots containing only soil but no plants. The combination of soil texture, plastic fragments (concentration or size) and watering resulted in 16 treatments for each experiment. All treatment combinations were applied to both planted and unplanted pots and were replicated ten times, resulting in 320 pots for each experiment. Pots were kept in a greenhouse at 20 °C with a day/night cycle of 10 and 14 h and a light intensity of 220 lux.

*Target species* We used the phytometer *Arabidopsis thaliana var. columbia* for both experiments. Seeds of *A. thaliana* were sown in standard greenhouse soil and cold-stratified at 8 °C for four days. Subsequently, they were moved to a climate chamber at 20–22 °C for 11 days, and when they reached an appropriate size (15 days after sowing), they were transplanted individually in assigned pots. Before planting, seedlings’ roots were quickly immersed in water to remove potential soil residues. During the first week after planting, any individual found dead was replaced. To minimize mortality, all pots were initially watered to saturation (i.e. 50 ml for pots with sand-rich soils and 60 ml for pots with clay-rich soils), and water treatments were imposed starting a week after transplanting. Plants received nutrients once a week (Wuxal^®^ Super, liquid 8-8-6 NPK fertilizer) initially at a concentration of 5 ml/l, and subsequently at a concentration of 1 ml/l.

*Measurements* All pots were weighed weekly, and the weight of unplanted pots was used as a proxy for soil water content. Measurements of plant diameter (cm) and height (cm) were taken weekly. Nine weeks after planting, we recorded the number of fruits produced and subsequently, we harvested plant above- and below-ground biomass (g). After washing the roots to remove soil and plastic fragments, all above- and below-ground biomass was oven dried at 70 °C for 48 h, and weighed on a high precision scale (Kern 770/GS/GJ Version 2.1). Radial growth (cm) was estimated as the difference between the initial plant diameter and diameter measured on the seventh week of measurements. Root-to-total mass ratio was calculated as the proportion of root to total biomass weight.

*Statistical analyses* We analyzed each experiment separately. We assessed correlations among response variables within each experiment using the R package *GGally*^51^. Then, we tested the effects of soil texture, plastic fragments (concentration or size), watering treatment and all two-way and three-way interactions applying linear models for aboveground biomass, root-to-total mass ratio, radial growth and plant height, and generalized linear models with negative binomial distribution for number of fruits, using the R package *MASS*^52^. Aboveground biomass was square-root transformed to fulfill model assumptions. We built a separate model for each response variable.

Because the differences in pot weight within each treatment were consistent across time, we used final pot weight (g) as a proxy for soil water content. Soil water content was analyzed using linear models, as a function of soil texture, plastic fragments (concentration or size), water treatment and all two-way and three-way interactions. To test whether patterns of soil water content were consistent in planted and non-planted pots (i.e. accounting for potential effects of plant roots on soil aggregation and porosity) we applied the same model to the final weight of planted pots. All significant interactions were assessed with Tukey HSD posthoc tests using the R package *emmeans*^53^. Data analysis and visualization were carried out in the software R v. 4.1.2^54^ and the packages *tidyverse*^55^, *patchwork*^56^, and *RColorBrewer*^57^.

### Experimental research and field studies on plants

The authors declare the experiment and the handling of plant material were carried out in compliance with relevant institutional, national, and international guidelines and legislation.

## Supplementary Information

Supplementary Information.

## Data Availability

Data are accessible on the Zenodo digital repository DOI 10.5281/zenodo.7225295.
