# Supplementary material for: Effects of plastic fragments on plant performance are mediated by soil properties and drought
Source: Sci Rep. 2022 Oct 22;12:17771. doi: 10.1038/s41598-022-22270-5 (PMC9588067; doi:10.1038/s41598-022-22270-5)

## Supplementary Information

# **Title:** **Effects of plastic fragment on plant performance are mediated by soil properties and drought**

**Authors**: Anne Krehl^1†^, Undine Schöllkopf^1†^, Maria Májeková^1^, Katja Tielbörger^1^, Sara Tomiolo^1*^

**^1^**Plant Ecology Group, Institute for Evolution and Ecology, Tübingen University, Auf der Morgenstelle 5, 72076 Tübingen, Germany

† These authors contributed equally to the study


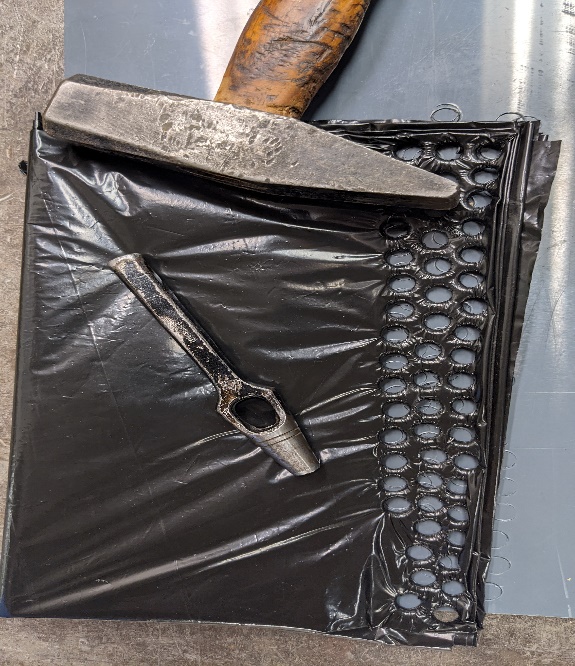
**Figure S1**: Photo showing how plastic fragments were produced. Mulching foil was folded and hollow punches of varying sizes (4 mm, 6mm,8 mm) were pushed with a hammer through the layers of foil.

**Figure S2: Plots showing mean ±1SE soil water content of planted pots, as a function of water treatment, soil texture and plastic fragments’ concentration (a) or size (b). Results of significant differences (p< 0.05) for pairwise comparisons of the effects of plastic fragments within each combination of soil texture and water treatment are reported with different letters. Semi-transparent dots represent the raw data. Lower pot weight represents a proxy for a lower soil water content.**


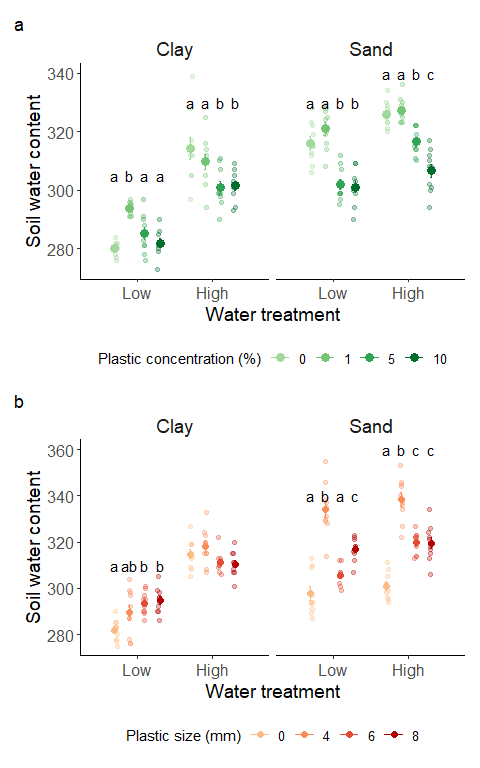


**Figure S3:** **Correlation matrix showing the correlation coefficients among all response variables for *Experiment 1* (a) and *Experiment 2* (b). Correlation coefficients indicated by the numbers in each cell are marked in blue when negative and in red when positive.**


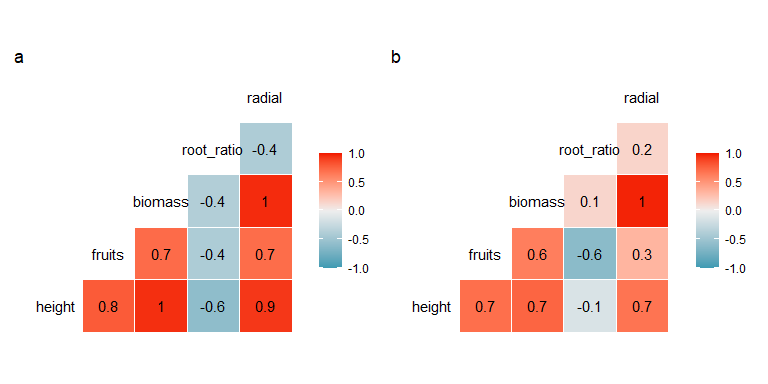


**Figure S4:** **Plot showing mean ±1SE radial growth of *A. thaliana* as a function of soil texture and plastic fragments’ size in a) low water treatments and b) high water treatments. Results of significant differences (p< 0.05) for pairwise comparisons of the effects of plastic fragments within each soil texture are reported with different letters Semi-transparent dots indicate the raw data**


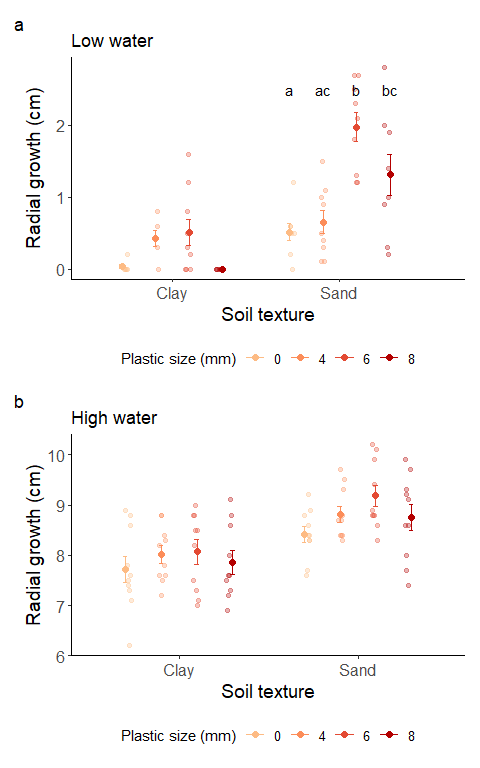


**Table S1:** results of linear models for *Experiment 1* testing the effects of water treatment (‘watering’), soil texture (‘soil’) and concentration of plastic fragments (‘plast_conc’) on soil water content of planted pots and the aboveground biomass of *A. thaliana* plants. Significant effects (p< 0.05) are reported in bold.

|  | **Soil water content** | | | **Aboveground biomass** | | | |
| --- | --- | --- | --- | --- | --- | --- | --- |
| **Model term** | **df** | **F.test** | **p.value** | **df** | **F.test** | | **p.value** |
| soil | 1 | 165.45 | **<0.001** | 1 | | 30.38 | **<0.001** |
| watering | 1 | 12.51 | **0.001** | 1 | | 595.14 | **<0.001** |
| plast_conc | 3 | 25.94 | **<0.001** | 3 | | 13.72 | **<0.001** |
| soil:watering | 1 | 38.00 | **<0.001** | 1 | | 7.65 | **0.006** |
| soil:plast_conc | 3 | 9.76 | **<0.001** | 3 | | 1.40 | 0.245 |
| watering:plast_conc | 3 | 2.15 | 0.097 | 3 | | 3.66 | **0.014** |
| soil:watering:plast_conc | 3 | 5.90 | **0.001** | 3 | | 1.04 | 0.379 |

**Table S2:** results of linear models for *Experiment 2* testing the effects of water treatment (‘watering’), soil texture (‘soil’) and size of plastic fragments (‘plast_size’) on soil water content of planted pots and the aboveground biomass of *A. thaliana* plants. Significant effects (p< 0.05) are reported in bold.

|  | **Soil water content** | | | **Aboveground biomass** | | |
| --- | --- | --- | --- | --- | --- | --- |
| **Model term** | **df** | **F.test** | **p.value** | **df** | **F.test** | **p.value** |
| soil | 1 | 19.79 | **<0.001** | 1 | 34.73 | **<0.001** |
| watering | 1 | 110.87 | **<0.001** | 1 | 2240.29 | **<0.001** |
| plast_size | 3 | 2.44 | 0.067 | 3 | 6.87 | **<0.001** |
| soil:watering | 1 | 45.18 | **<0.001** | 1 | 0.74 | 0.392 |
| soil:plast_size | 3 | 21.14 | **<0.001** | 3 | 3.13 | **0.028** |
| watering:plast_size | 3 | 6.97 | **<0.001** | 3 | 3.37 | **0.021** |
| soil:watering:plastic_size | 3 | 6.99 | **<0.001** | 3 | 1.25 | 0.294 |

### Appendix 1: results for the effects of water treatment, soil texture and plastic concentration (*Experiment 1*) or plastic size (*Experiment 2*) on the soil water content of planted pots and the aboveground biomass, root-to-total mass ratio, height, and fruit production of *A. thaliana* plants growth

*Experiment 1:*

*Final height*: plants grew significantly taller in high water treatments (F_1_ = 226.86, p =<0.001), while no other factor showed significant effects. In clay-rich soils exposed to low water treatments and to plastic concentrations 0% and 0.1% plants remained flat and did not grow vertically (Fig. S5a).  *Root to total biomass ratio:* we found a significant effect of soil texture (F_1_ = 19.19, p<0.001) and a significant interaction between soil texture water treatment (F_1_ = 11.52, p <0.001), indicating a higher production of roots in low compared to high water treatments in clay-rich soil, but no significant differences in sand-rich soil (Fig. S5b). *Aboveground biomass:* we found significant two-way interactions between soil texture and water treatment and between water treatment and plastic fragments (Table S1). The high water treatment had a significant positive effect on plant biomass, and the difference in biomass across water treatments was slightly higher in clay-rich soil. Plants attained the highest biomass in the highest plastic concentration (1%) (Fig. S5c). *Number of fruits:* Plants produced fruits only in high water treatments. Although, we found a trend of increasing number of fruits with higher concentration of plastic fragments, neither soil texture or plastic concentration had a significant effect (Fig. S7a).

### *Experiment 2*

### *Final height:* hardly any plant grew vertically in low water treatments (Fig. S6a). Plants grew significantly taller in sand-rich compared to clay-rich soil (F_1_ = 7.92, p = 0.006). *Root to total ratio:* the significant interaction between soil texture and water treatment (F_1_ = 6.46, p =0.01) indicated higher root to total biomass ratio in high water compared to low water treatments in sand-rich soil, while no differences were found in clay-rich soils (Fig. S6b), a pattern opposite to that found in *Experiment 1*. *Aboveground biomass:* we found significant two-way interactions between soil texture and watering and between watering and plastic fragments (Table S2). The high water treatment affected plant biomass positively, and this effect was stronger in sand-rich soil. Plants attained the highest biomass in the highest plastic size (8 mm) (Fig. S6c).

### *Number of fruits:* plants produced fruits only in high water treatments, with a higher production of fruits in sand-rich compared to clay-rich soil (df =1, Chisq = 27.75, p<0.001), no significant effect of plastic fragments’ size was detected (Fig S7b)

###

**Fig S5: plots showing mean ±1SE a) plant height, b) root-to-total biomass ratio, c) aboveground biomass as a function of water treatment, soil texture and plastic fragments’ concentration. Semi-transparent dots represent the raw data. Results of significant differences (p< 0.05) for pairwise comparisons of the effects of plastic fragments within each combination of soil texture and water treatment are reported with different letters.**


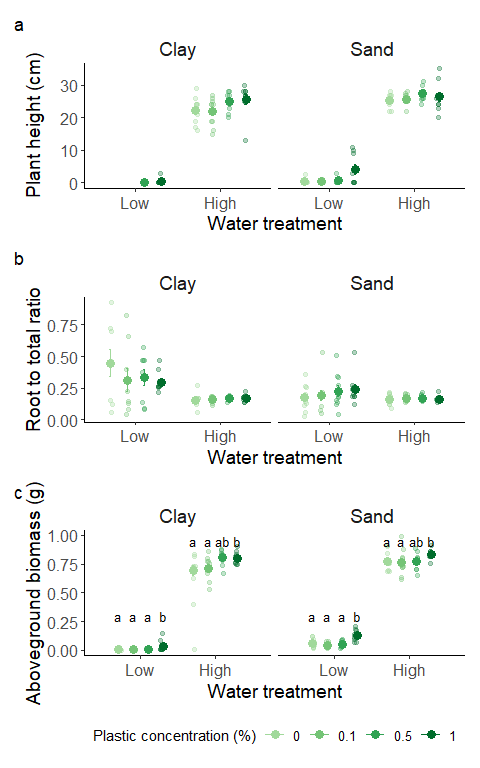


**Fig S6: plots showing mean ±1SEa) plant height, b) root-to-total biomass ratio, c) aboveground biomass as a function of watering treatment, soil texture and plastic fragments’ size (b). Semi-transparent dots represent the raw data. Results of significant differences (p< 0.05) for pairwise comparisons of the effects of plastic fragments within each combination of soil texture and water treatment are reported with different letters.**


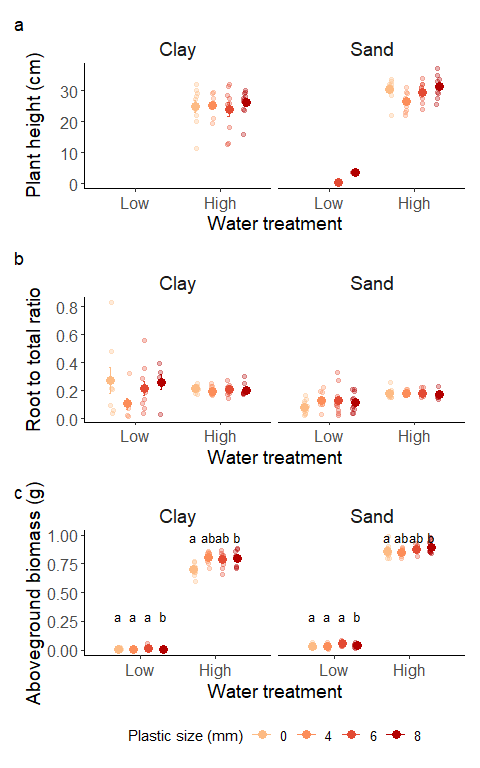


**Fig. S7: plots showing mean +/-1SE number of fruits in high water treatments as a function soil texture and plastic fragments’ concentration (a) or plastic fragments’ size (b). Semi-transparent dots represent the raw data.**


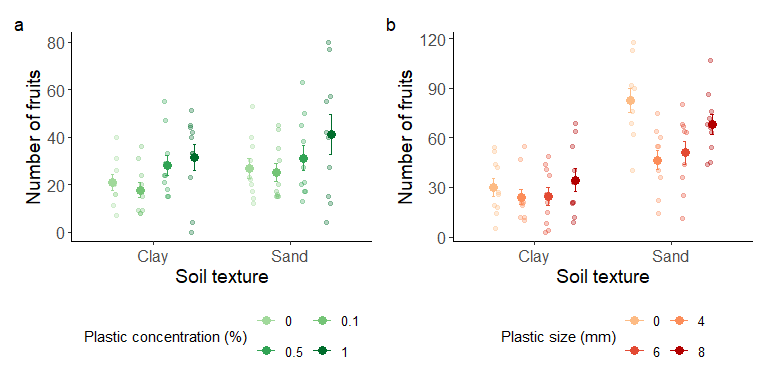

Supplement: Supplementary file 1 — Supplementary Information. [file 41598_2022_22270_MOESM1_ESM.docx]
